# Macrophages as Potential Therapeutic Targets in Acute Myeloid Leukemia

**DOI:** 10.3390/biomedicines12102306

**Published:** 2024-10-11

**Authors:** Oana Mesaros, Madalina Onciul, Emilia Matei, Corina Joldes, Laura Jimbu, Alexandra Neaga, Oana Serban, Mihnea Zdrenghea, Ana Maria Nanut

**Affiliations:** 1 Department of Hematology, Iuliu Hatieganu University of Medicine and Pharmacy, 8 Babes Str., 400012 Cluj-Napoca, Romania; 2 Department of Hematology, Ion Chiricuta Oncology Institute, 34-36 Republicii Str., 400015 Cluj-Napoca, Romania; 3 Department of Pathology, Ion Chiricuta Oncology Institute, 34-36 Republicii Str., 400015 Cluj-Napoca, Romania; 4 Octavian Fodor” Regional Institute of Gastroenterology and Hepatology, 19-21 Croitorilor Str., 400162 Cluj-Napoca, Romania; 5 Regina Maria” Regional Laboratory in Cluj-Napoca, 109 Observatorului Str., 400363 Cluj-Napoca, Romania; 6 Regina Maria” Regional Laboratory in Cluj-Napoca, 34-36 Republicii Str., 400015 Cluj-Napoca, Romania

**Keywords:** therapeutic targets, leukemia-associated macrophages, tumor-associated macrophages, acute myeloid leukemia

## Abstract

Acute myeloid leukemia (AML) is a heterogenous malignant hemopathy, and although new drugs have emerged recently, current treatment options still show limited efficacy. Therapy resistance remains a major concern due to its contribution to treatment failure, disease relapse, and increased mortality among patients. The underlying mechanisms of resistance to therapy are not fully understood, and it is crucial to address this challenge to improve therapy. Macrophages are immune cells found within the bone marrow microenvironment (BMME), of critical importance for leukemia development and progression. One defining feature of macrophages is their plasticity, which allows them to adapt to the variations in the microenvironment. While this adaptability is advantageous during wound healing, it can also be exploited in cancer scenarios. Thus, clinical and preclinical investigations that target macrophages as a therapeutic strategy appear promising. Existing research indicates that targeting macrophages could enhance the effectiveness of current AML treatments. This review addresses the importance of macrophages as therapeutic targets including relevant drugs investigated in clinical trials such as pexidartinib, magrolimab or bexmarilimab, but also provides new insights into lesser-known therapies, like macrophage receptor with a collagenous structure (MACRO) inhibitors and Toll-like receptor (TLR) agonists.

## 1. Introduction

Acute myeloid leukemia (AML) is a heterogeneous cancer marked by the rapid increase in clonal myeloid progenitors (blasts) in the bone marrow and/or peripheral blood. Currently, the AML curability rate does not exceed 50% even for patients younger than 60 years [1], who are typically able to withstand intensive treatment approaches. Macrophages are phagocytic cells that are vital for the physiological functioning of innate immunity. The various functions of macrophages are related to their plasticity, a feature that characterizes macrophages and allows them to adapt to the changes within the tumor microenvironment (TME). In response to TME stimuli, macrophages can polarize to different forms, notably M1 and M2 macrophages. In response to the action of extrinsic factors in the tissue microenvironment, macrophages initiate various intracellular pathways that trigger their development towards distinct phenotypes. Apart from their immune role, a certain subtype of macrophages manifests a protective and nurturing effect, favoring neoplastic cells. These are called tumor-associated macrophages (TAMs) [2]. As a response to an unhealthy malignant microenvironment, TAMs become activated and switch to specific phenotypes, participating in and supporting tumor progression. They are found in the TME of various leukemia types, where they are referred to as leukemia-associated macrophages (LAMs) and are linked to disease progression [3].

LAMs reside within the bone marrow microenvironment (BMME), which is formed of different cell types, such as osteoclasts, stromal cells, vascular endothelial cells, and immune effectors like natural killer (NK) cells [4]. LAMs are crucial components of the TME and they influence tumor progression. They produce a range of cytokines, chemokines, and proteases that can facilitate immunosuppression and induce tumor growth and metastasis [5].

This work focuses on LAMs as targets for therapy, reviewing the most recent research and clinical trials. We will start by discussing the functions of macrophages, including the currently accepted paradigm of their polarization. Next, we will address the pro-tumorigenic role of TAMs in neoplastic disease and focus on LAMs’ relevance in AML. The final section is dedicated to past and present attempts at using macrophages as therapeutic targets in AML.

## 2. The Functions of Macrophages

In the 19th century, Ilya Metchnikoff was the first scientist who studied macrophages, and later, in 1960, Van Fürth noted that all macrophages originate from circulating blood monocytes [6,7,8,9,10]. However, recent findings revealed that his theory was only partially true, as some macrophages are present from embryogenesis and continue to exist in tissues, regardless of monocytes [11,12,13,14,15,16,17].

Macrophages are phagocytic cells, involved in innate immunity. Additionally, they participate in adaptative immunity by initiating specific defense mechanisms and recruiting immune cells, including lymphocytes [18]. Macrophages are known as antigen-presenting cells (APCs) because they protect against pathogens by recognizing and phagocytizing non-self-organisms, delivering the antigens to adaptive immune cells. Once activated, macrophages secrete cytokines and chemokines that initiate the process known as inflammation and influence the microenvironment [19,20]. Inflammation is a physiological reaction to infections or injuries, including microbial attacks or trauma. It is essential for maintaining homeostasis and is a highly regulated process comprising pro-inflammatory and anti-inflammatory elements [21]. During the initial phases of inflammation, macrophages destroy abnormal cells and remove apoptotic bodies [22].

When macrophages engulf pathogens, they process the antigens and display them on the surface of their membrane. This allows B cells and T-helper cells to recognize these antigens, particularly those encoded by major histocompatibility complex II (MHC II). After antigen recognition, T cells produce cytokines to stimulate B cells, which will release antibodies specific to the antigen [23]. Macrophages have increased heterogeneity due to their diversity of surface receptors [24].

Alongside neutrophils, macrophages serve as the primary defense mechanism against pathogen invasion. However, their phagocytic ability decreases with aging [25,26]. When macrophages are exposed to inflammatory stimuli, they secret cytokines such as tumor necrosis factor (TNF), inflammation-associated interleukins such as IL-1, IL-6, IL-8, and IL-12, chemokines, leukotrienes, and prostaglandins, which can enhance vascular permeability and attract inflammatory cells, thereby promoting and maintaining inflammation [27]. In addition to producing pro-inflammatory proteins, macrophages also release anti-inflammatory proteins designed to mitigate the immune response against pathogens, as an excessive inflammatory reaction could lead to self-tissue damage. Based on these findings, the currently accepted paradigm categorizes macrophages into two subtypes: pro-inflammatory or M1 subtype and anti-inflammatory or M2 subtype [28].

M2-activated macrophages release anti-inflammatory cytokines that help regulate M1 pro-inflammatory macrophages, reduce inflammatory responses, and promote injury recovery and tissue restoration [29]. While the macrophages’ plasticity is advantageous for wound healing, their response becomes altered in the presence of tumors. The TME alters and reconfigures the immune response, facilitating and maintaining tumor growth.

## 3. Activation of Macrophages

Macrophages have several functions that help maintain immune homeostasis, including tissue repair, phagocytosis, clearance of foreign materials, and the secretion of cytokines and complement proteins. These processes are essential for immune responses to infections and are involved in inflammation. To carry out their protective roles and repair damaged tissues, macrophages display a wide range of surface, vacuolar, and cytosolic receptors, which will facilitate the phagocytosis and endocytosis of viral and bacterial components [4].

Macrophages exhibit a diverse range of functions and characteristics. Their activation into either M1 or M2 phenotypes is known as “polarization”, a biological process that occurs in response to signals received from the surrounding microenvironment [30,31]. This is a crucial process for tissue repair and maintaining homeostasis [28]. M1 polarization is referred to as the “classical” pathway, whereas that of M2 is identified as the “alternative” pathway [32]. M1 macrophages secrete pro-inflammatory cytokines, such as TNF, IL-12, and gamma interferon (IFN-γ), while also producing high amounts of nitric oxide synthase (NOS), which is responsible for converting arginine into nitric oxide (NO) [33,34].

In the presence of lipopolysaccharides (LPSs), macrophages switch their phenotype towards M1 and upregulate CD80, CD86, Toll-like receptor (TLR)-2, and TLR-4 expression. M1 macrophages are controlled by nuclear factor-kappa B (NF-ҡB), signal transducer and activator of transcription (STAT) (specifically STAT1 and STAT5), and interferon regulatory factor (IRF) family members, including IRF3 and IRF5. The first two represent the primary pathways that drive macrophage polarization, enhancing their antimicrobial and antitumoral functions [18,35,36]. M2 macrophages are CD163-, CD209-, and CD206-positive cells [4,37]. This macrophage subtype is primarily regulated by the STAT6 pathway, IRF4, and peroxisome proliferator-activated receptor gamma (PPAR-γ). STAT6 is considered the most important among them [38].

Although macrophages are dichotomized as M1 and M2, they are closely interconnected. The regulation of macrophage polarization involves various factors, including the STAT family, PPAR-ỿ, the cAMP-responsive element-binding protein (CREB)-CCAAT/ enhancer-binding protein (C/EBP) axis, IRF, and NF-ҡ B family. This process encompasses numerous signaling molecules, such as JAK/STAT and c- Jun N- terminal kinase (JNK) [39,40]. Table 1 presents the macrophages’ activation and polarization. The M1 and M2 pathways have antithetical functions. M2 macrophages promote cell repair and growth by secreting ornithine, whereas M1 macrophages drive destruction through NO production, which is involved in microbicidal activity and the inhibition of cell proliferation. An imbalance between these two macrophage types can result in metabolic instability, potentially leading to the onset of autoimmune diseases or even cancer [41].

The term “alternative activation” has been extended to include new phenotypes, like M2 a/b/c/d [42]. The M2a phenotype is produced in vitro by exposing cells to IL-4 or IL-13, resulting in enhanced levels of CD206, arginase, and transforming growth factor β (TGF-β). Meanwhile, the M2b phenotype is triggered by several IgG and LPS immune complexes, which induce the production of IL-10, while lowering IL-12 levels [43,44]. Macrophages that were exposed in vitro to IL-10 or glucocorticoids induce the M2c phenotype, characterized by increased IL-10, reduced IL-12 levels, and enhanced CD163 surface receptor expression. In contrast, M2d-like macrophages are activated by Toll-like receptors (TLRs) and stimulate IL-10, vascular endothelial growth factor (VEGF), and epidermal growth factor (EGF) production, thus aiding angiogenesis and tumoral progression. Additionally, fibroblast growth factor (FGF) and platelet-derived growth factor (PDGF) can facilitate tumor cell development and metastasis, in various cancers, as well as in AML [45,46].

In summary, macrophages can adopt various phenotypes in reaction to different stimuli.

## 4. Tumor-Associated Macrophages

The emergence of various cancer types, including leukemia, is not solely due to genetic and epigenetic alterations; it also involves pathological changes in the microenvironment, including the stroma and its cellular components [47]. For instance, tumor cells can evade immune responses, causing stromal and immune cells—typically responsible for defending the host against harmful threats—to secrete anti-inflammatory cytokines. These cytokines ultimately protect the tumor cells and facilitate their growth and proliferation [48,49]. As previously noted, macrophages that invade tumor tissues or the TME are referred to as TAMs. TAMs significantly contribute to tumor progression by fostering genetic instability and metastasis, and they also participate in the suppression of adaptive immunity [50]. Most TAMs are present in avascular areas [51,52].

In individuals diagnosed with breast, ovarian, or prostate cancers, TAMs are elevated, particularly of the M2 subtype, which has been associated with faster tumor progression, metastasis, and reduced response to treatment. Conversely, in lung cancer, a higher presence of M1 macrophages has been associated with more favorable outcomes due to their capacity to hinder tumor progression and enhance the effects of chemotherapy [53]. In colorectal cancer, TAMs seem to exhibit controversial roles. Certain research indicates that a higher presence of macrophages may be linked to improved outcomes, whereas other findings suggest the opposite. One possible explanation for this discrepancy is that TAMs can be found both within and outside the tumor, and their functions may vary based on their location [54]. Recent investigations revealed that M2 macrophages are more prevalent than the M1 subtype in lung tumors, while M1 macrophages are predominant in colon carcinomas. In the context of lung cancer, M2 macrophages facilitate cell invasion and tumor development, unlike M1 macrophages, which inhibit cell growth, decrease angiogenesis, and trigger apoptosis in lung cancer cells [55,56,57,58].

LAMs/TAMs’ phenotypic and functional traits differ based on the unique microenvironments found in different organs [57]. It remains unclear which factors contribute to altering macrophage polarization, promoting the growth of cancerous cells rather than suppressing their proliferation [59]. When TAMs are polarized as the M1 subtype, they function as pro-inflammatory and antitumor cells. In contrast, a transition to the M2 phenotype reveals tumor-nurturing attributes; their presence is linked to a poor prognosis [60]. The development of pro-tumorigenic TAMs depends on elements that promote macrophage expansion as well as factors that trigger their polarization [61]. The infiltration of macrophages in the TME is regulated by CCL2, CCL5, CCL7 and CX3CL1 chemokines, in addition to VEGF [62,63].

In the TME, there are interactions among TAMs and cancer and stromal cells. Factors secreted by the tumor cells, including IL4, IL10, and stromal-derived factors, lead to a transition towards the M2 subtype and attract new macrophages and inflammatory cells [64,65].

After recruitment and polarization, TAMs can contribute to tumor progression, metastasis, resistance to chemotherapy, and immune evasion [66]. Once macrophages accumulate in the TME, several cytokines produced by the tumor or stromal cells initiate the tumor-nurturing transformation of TAMs. IL-4 and IL-13 act on TAMs through STAT6 and phosphoinositide-3-kinase (PI3K) pathways. Additionally, IL-10 released from Tregs promotes the M2 polarization of TAMs via STAT3 activation. Factors secreted by macrophages that contribute to tumor progression include ornithine, VEGF, EGF, and TGF-β. In contrast, NO generated from ROS within macrophages can inhibit tumor growth [67].

**Figure 1:**
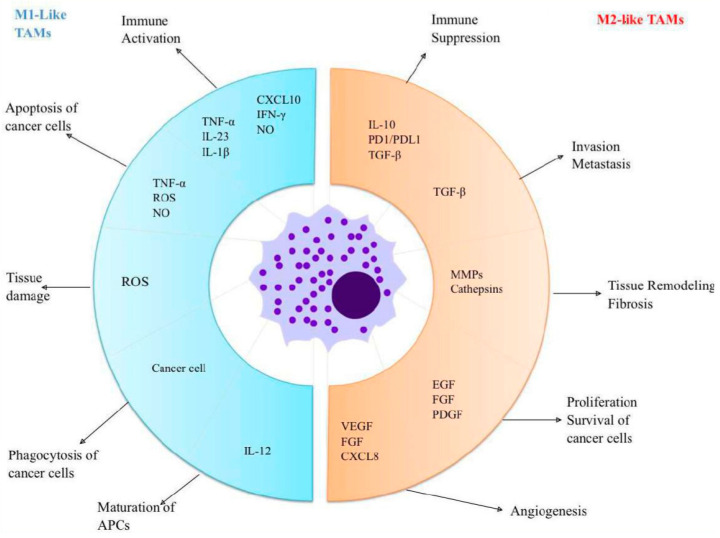
TAMs and their functions: M1-like macrophages have antitumor effects and are activated by inflammatory cytokines. These macrophages produce chemokines like CXCL10, which play a crucial role in attracting and activating T cells. Additionally, M1-like macrophages actively phagocytose cancer cells while releasing TNF-α, ROS, and NO to target and eliminate neoplastic cells. In contrast, M2-like macrophages serve pro-tumor roles; they secrete factors that enhance and promote tumor growth. Furthermore, they produce immune-suppressive substances, which support the function of regulatory T cells. Abbreviations: TAMs = Tumour-associated macrophages; APCs = Antigen-presenting cells; TNF = Tumor necrosis factor; IL = Interleukin; CXCL = Chemokine (C-X-C motif) ligand; INF = Interferon; NO = Nitrogen oxide; ROS = Reactive oxygen species; PD = Programmed death; PDL = Programmed death ligand; TGF = Transforming growth factors; MMPs = Matrix metalloproteinases; EGF = Epidermal growth factor; FGF=Fibroblast growth factor; PDGF = Platelet-derived growth factor; VEGF = Vascular endothelial growth factor.

As mentioned above, the polarization of TAMs is induced by several cytokines, chemokines, growth factors, and tumor and stromal cells [68]. Colony-stimulating factor (CSF-1) and CC motif ligand 2 (CCL2) represent some of the most extensively researched factors that stimulate M2-type macrophages [69]. CSF-1 plays a critical role in facilitating the harvesting of monocytes from the peripheral blood, guiding them through their maturation process, and pushing their polarization towards the M2 subtype, through its interaction with the CSF-1R receptor. CSF-R1 signaling pathways are essential for controlling the differentiation, proliferation, and survival of macrophages and their precursors, monocytes. Recent studies indicate that signaling through CSF-1/1R within tumors may enhance TAM2’s recruitment and foster an anti-inflammatory environment, ultimately aiding in tumor progression and metastasis. CSF-1 exhibits both autocrine and paracrine effects within the tumor milieu, underscoring its involvement in tumor growth. CCL2 facilitates the polarization of macrophages towards the M2 phenotype, which is associated with tumor growth stimulation by engaging the CC2 chemokine receptor (CCR2) from the macrophage surface [68]. In tumor-bearing mouse models, blocking the CCL2-CCR2 interaction led to extended survival and reduced levels of pro-tumorigenic cytokines [4,70,71]. Figure 1 presents TAMs and their functions.

Several studies indicate that TAMs can drive cancer cells to develop treatment resistance by enhancing the properties of cancer stem cells (CSCs), which play a role in the emergence of tumor angiogenesis [72,73,74,75]. Furthermore, TAMs produce cytokines and chemokines, including IL-6 and CCL18, which contribute to the development of therapeutic resistance [54]. TAMs induce resistance to immunotherapy by impeding T-cell functions. Within the TME, TAMs interact with other cells, promoting tumor growth and contributing to chemoresistance. Despite their various roles in the microenvironments of hematological malignancies, the significance of TAMs as diagnostic and potentially prognostic markers is still not fully understood, although emerging studies indicate promising findings [76,77]. While TAMs in the TME appear to facilitate tumor proliferation and progression, their mechanisms of action may differ depending on the specific disease context [40,67].

Chimeric antigen receptor T cells (CAR-Ts) are a form of cellular therapy that was FDA-approved for hematological malignancy treatment. However, the absence of indications for this type of therapy in AML and other solid tumors led to the development of CAR–natural killer cells (CAR-NKs) and CAR–macrophage cells (CAR-Ms) [78]. CAR-Ms are developed to enhance the macrophages’ capacity to trigger innate immunity and phagocytize tumor cells. In 2020, the first clinical trial employing CAR-Ms as a therapy for HER2-positive solid tumors was launched. Further, the technology was developed to address lymphoproliferative disorders as well, with a good safety profile [79]. However, to date, no efficacy of CAR-Ms in AML has been proven.

## 5. M2 Macrophages in AML

In the context of AML, LAMs are essential contributors to therapeutic resistance and leukemia cells’ protection from apoptosis induced by cytarabine [5]. The main goal of macrophage-targeted therapy in neoplastic disease is to reduce the population of anti-inflammatory macrophages while enhancing the presence of pro-inflammatory macrophages that support antitumor activity [80].

Macrophage invasion in the TME has been recognized as a poor prognostic indicator in various cancers, including leukemias [81]. There is growing interest in immunotherapies that target TAMs/LAMs [80]. These therapies inhibit their pro-tumor signaling, restore their immune-stimulating functions, and deplete TAMs [74,82]. With an enhanced understanding of the role of macrophages in hematological cancers, potential strategies for targeting LAMs have been proposed and investigated for therapeutic use [83].

The PI3K-AKT-mTOR signaling pathway shows abnormal regulation in individuals diagnosed with AML, as a result of various molecular mutations, including FTL3-ITD. This alteration is present in almost 30% of AML patients and has been linked to poorer survival outcomes [84,85]. Class I PI3K is made up of the p85 regulatory subunit and the four isoforms of the p110 subunits: α, β, γ, and δ. AML blasts consistently express the p110 δ isoform [86]. Targeting both PI3K γ and PI3K δ with the selective inhibitor IPI-145 has shown a decrease in AML blast survival, secondary to the inhibition of AKT survival pathways, and also by affecting macrophage polarization [87,88]. mTOR signaling is essential for macrophage polarization, and research showed that the inhibition of mTORC2 stimulates the shift of macrophages towards the M2 subtype, whereas the mTORC1 blockade enhances M1 macrophage function [89,90]. However, mTOR inhibitors did not show spectacular results in AML treatment in clinical and preclinical studies [91,92].

Rather than using macrophages as direct targets, focusing on their re-education seems a good therapeutic strategy. This is supported by Mussai et al., who proved that the production of arginase II by AML blasts induces the reprogramming of immunocompetent donor monocytes into the anti-inflammatory macrophage subtype [93]. Bone marrow samples of AML patients were assessed by immunohistochemical analysis that revealed an enhanced CD206+ cell level, which are associated with M2 polarization of macrophages [94]. Research conducted by AL-Matary et al. employed various mouse models of AML, including transgenic models featuring mutations like NUP98-HOXD13. Their findings indicated that M2-like macrophage levels were elevated in the bone marrow of leukemic mouse models compared to those without leukemia. In particular, studies on the NUP98-HOXD13 mouse model, which mimics t(2;11)(q31;p15), revealed a negative correlation between the elevated percentage of alternatively activated macrophages (AAMs) in these mice and their survival rates [95]. Among AML patients, researchers have noticed a reduction in the amount of monocytic leukemia zinc finger protein (MOZ), which plays a role in the macrophage development cycle, by interacting with miR-223. In contrast, miR-223 levels were significantly elevated when compared to those in clinically and biologically healthy individuals. Furthermore, actual research correlated low MOZ expression with the monocytic subtype of AML [95].

The precise macrophage function in resistance to treatment remains incompletely understood. Macrophages secrete substances that activate the extracellular signal-regulated kinase pathways 1 and 2 (ERK1/2) and regulate MCL-1 protein expression [96].

M2-like macrophages consistently produce increased amounts of CCL2. Research has shown that in response to its influence, AML blasts will migrate due to their expression of functional CCR2 [97]. CCL2 is considered a prognostic indicator for AML, as elevated CCL2 levels in untreated AML patients were associated with poor cytogenetic profiles and, thus, with an unfavorable prognosis [98,99,100].

In AML patients, high serum levels of chitinase-3-like (CHI3L) were linked to a poor prognosis [101,102]. In ovarian cancer, CHI3L1 plays a role in activating the extracellular signal-regulated kinase (ERK) 1/2 pathway, which enhances resistance to chemotherapy by increasing the level of MCL-1 protein [103]. Additionally, research indicates a significant relationship between CHI3L and the M2 polarization of macrophages [104].

## 6. Therapeutic Targets of Macrophages

The standard treatment for young and fit patients with AML who can handle intensive therapy is the “3 + 7” regimen, which combines an anthracycline with cytarabine. However, the current results of this treatment regimen are not entirely satisfying. Immunotherapy presents a promising alternative for managing both solid and blood tumors. It may provide a less aggressive and better-tolerated option for treating AML, with the potential to elicit more durable responses and improve life expectancy [105].

AML cells can establish mechanisms to effectively evade cell death, such as inhibiting NK cells or downregulating specific surface receptors. Concurrently, they enhance the expression of inhibitory immune checkpoints, such as programmed death ligands (PD-L1 and PD-L2), CD47 and CD 70 [106,107,108].

Under physiological circumstances, programmed cell death protein 1 (PD-1) is present on activated T cells and B cells as well as regulatory T cells (Tregs). PD-1 acts to prevent excessive immune activation by binding to its receptors, PD-L1 and PD-L2, expressed on macrophages and dendritic cells. This interaction generally allows tumors to evade the immune response by inhibiting T-cell activity and blocking cytokine signaling, while simultaneously providing an anti-apoptotic signal to tumor cells via PD-L1. Consequently, PD-1/PD-L1/PD-L2 inhibitors—known as checkpoint inhibitors—have shown substantial efficacy in treating Hodgkin’s lymphoma, although their impact in AML treatment was considerably lower [109,110]. The differences between M1 and M2 subsets of macrophages may be associated with important factors, including disease severity and the timing of diagnosis. The presence of high levels of the anti-inflammatory macrophage subtype in the bone marrow of patients may be related to their disease evolution and response to checkpoint inhibitors, potentially serving as a prognostic indicator [94,97]. Modulating M2 macrophage expression could improve PD-1/PD-L1 inhibitor treatment outcomes in AML [111].

Yang et al. demonstrated that AML patients with high CD163 expression levels experienced lower survival rates than those with reduced levels of CD163. Additionally, CD163 is involved in characterizing M2-like macrophages [40]. Another study examined the immunophenotypic characteristics of sixteen AML patients and identified a link between CD163-positive macrophages and unfavorable outcomes. Conversely, the existence M1-like macrophages in the TME of AML suggests a more favorable prognosis. The balance between M1 and M2 macrophages could be clinically significant for individuals with AML [112]. The M2 anti-inflammatory macrophages within the TME are typically correlated with a poor prognosis, as patients exhibiting elevated levels of M2 macrophages tend to have reduced survival [72,113].

The cells within the TME, particularly LAMs, are vital targets for improving the efficacy of immunotherapy, as they have the ability to suppress the cytotoxic functions of CD8+ T cells and NK cells. Eliminating LAMs could boost the antitumor immune response driven by CD8+ T cells [114].

Therapeutic approaches aimed at M2 macrophages in AML include blocking the recruitment of CCR2+CD14++ CD16- (TAM precursors), the inhibition of CCL2-CCR2 signaling pathways, and reprogramming M1-like macrophages in terms of their function and phenotype [115].

Currently, selective inhibitors of the p110 delta PI3K isoform are undergoing preclinical development and show promising results for patients with AML. These inhibitors have the ability to reduce macrophage invasion into tumors and shift macrophages towards an M1-like phenotype, but may also have an impact on AML blasts [88].

Another therapeutic approach involves the depletion of TAMs. The cytokine CSF-1 plays an essential role in the resilience and evolution of phagocytic cells that express the CSF-R1 receptor. Increased CSF-1 levels or overexpression of CSF-1R have been associated with unfavorable outcomes in Hodgkin’s lymphoma and hepatocellular carcinoma [116]. The pharmacological inhibition of the CSF-1/CSF-1R pathway has been investigated in both preclinical and clinical settings, whether used alone or in combination with other therapies. Targeting CSF-1R to reduce macrophages has led to increased migration of CD8+ cytotoxic T cells and improved therapeutic responses in breast and ovarian cancers [117,118].

CSF-1R targeted therapies are employed to prevent the dimerization of the receptors and decrease the survival of macrophages. Research in both clinical and preclinical studies has proven that modulating the CSF/CSF-1R pathway offers a promising avenue for therapy. CSF-1R inhibition leads to better outcomes for patients with advanced cancer and enhances the effectiveness of immunotherapy and chemotherapy [119]. Pexidartinib (PLX3397) is the most extensively researched compound, functioning as an oral inhibitor of the CSF-1R tyrosine kinase. One study indicated that administering PLX3397 to mouse models with breast tumors resulted in a significant reduction in macrophages and tumor development inhibition [120].

In a phase-1 study, PLX3397 could be administered alongside other treatments, such as binimetinib, which is indicated for gastrointestinal stromal tumors, and paclitaxel, indicated in advanced ovarian cancer treatment. These associations show significant clinical effectiveness and are generally well tolerated by patients [121,122]. Although depletion of TAMs seems to be a promising strategy for many types of cancer, it should be used selectively, as long-term administration can have detrimental effects. Prolonged inhibition of CSF-1R leads to resistance development and the recurrence of tumors [116].

Another CSF-1R inhibitor is edicotinib, which is used in prostate cancer therapy, without significant alterations in the CSF-1R+ immune cell population [123]. In 2021, a phase-two clinical trial was initiated for patients with relapsed/refractory AML receiving edicotinib. As only three patients were enrolled, more data are required to conclude whether this CSF-1R inhibitor is a relevant treatment option for AML [124].

Trabectedin and bisphosphonates promote apoptosis and can induce macrophage depletion. Trabectedin, an antineoplastic agent used for ovarian cancer, initiates apoptosis by binding DNA, resulting in cell cycle inhibition and the cleavage of double-stranded DNA [125,126].

Mononuclear phagocytes are vulnerable to recombinant TNF-related apoptosis-inducing ligand (TRAIL) through a caspase-dependent apoptotic pathway. Monocytes and macrophages exhibit highly expressed TRAIL receptors (TRAIL-R1 and TRAIL-R2), making them more responsive to trabectedin administration [127].

Studies have described trabectedin as an inducer of caspase-8-dependent apoptosis in TAMs, leading to a selective reduction in monocytes and macrophages in blood circulation and the TME. In biopsy samples from soft tissue sarcoma patients treated with trabectedin, a significant decrease in the density of TAMs and blood vessels was observed [128].

Bisphosphonates are absorbed by bone tissue and metabolized by osteoclasts. Since macrophages share the same cell lineage as osteoclasts, they are also targeted by bisphosphonates. These substances exhibit an antitumor effect by inducing apoptosis in tumor cells, boosting immune surveillance through macrophage targeting, inhibiting tumor cell invasion, reducing angiogenesis, and showing synergistic effects when used alongside other cancer treatments [129,130,131].

Another therapeutic strategy in AML leverages macrophages by targeting CD47, a protein that hinders phagocytosis by targeting the α receptor signaling protein on phagocytic cells. CD47 is overexpressed in AML and other malignant hemopathies. Monoclonal antibodies directed against CD47 enable macrophages to engulf and eliminate the AML cells [132,133].

**Figure 2:**
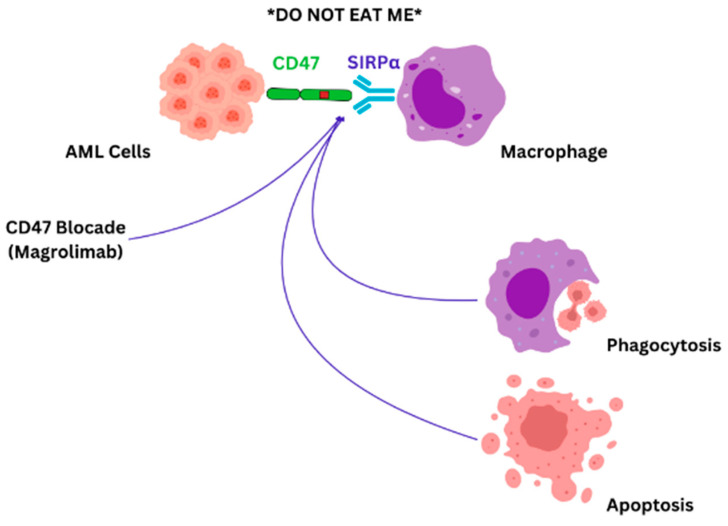
CD47-SIRPα blockade: AML cells evade immune responses by CD47-SIRPα synergy, which provides a “do not eat me” signal. Blocking CD47-SIRPα interaction with magrolimab will induce direct tumor cell apoptosis, complement-mediated apoptosis, and immune cell phagocytosis [79].

In AML, leukemic cells exhibit CD47 on their surface. Elevated levels of CD47 expression in patients with AML appear to correlate with a poor prognosis. Additionally, this heightened expression is particularly evident in AML patients carrying the FLT3 mutation, which is also linked to a worse prognosis [107]. The presence of CD47 on cell surfaces sends an inhibitory signal to macrophages, effectively signaling “do not eat”, which allows cancer cells to dodge immune elimination. As malignant cells express CD47 across various blood-related disorders, numerous anti-CD47 antibodies are under investigation to significantly enhance existing treatment options [134,135,136]. The “do not eat” signal is mediated by signal regulatory protein alpha (SIRPα) found on immune cells [134]. Magrolimab is a monoclonal antibody that targets CD47 and blocks the “don’t eat me” signal exerted by the leukemic cells (Figure 2).

Initially, magrolimab was evaluated in a phase-I multicentric trial as a standalone AML treatment. Hypomethylating agents were administered synergically to magrolimab, to induce “eat me” signals in the AML cells, thus stimulating the phagocytic process. The pairing of magrolimab and azacitidine has demonstrated promising efficacy in treating AML and myelodysplastic syndrome (MDS) [137]. In patients with AML harboring TP53 mutations, available treatment options are limited, and the outlook is poor [138]. In a phase-IB trial, the researchers assessed the effectiveness and tolerability of combining magrolimab with the hypomethylating agent azacitidine in 72 patients with AML who have the TP53 mutation [139]. The results were promising for this combination, with a good safety profile [139]. Additionally, the combination of magrolimab with the “3 + 7” regimen of low-dose cytarabine in treatment-naïve AML patients was shown to enhance clinical response rates [140]. Nevertheless, the effects of cytotoxic agents need to be closely monitored, as they may non-specifically affect pro-phagocytic signals in normal cells as well as leukemic cells, potentially resulting in toxicities [134,137,138,139,140,141]. Another agent that promotes cell death and may work synergistically with magrolimab is venetoclax [142]. It can convey pro-phagocytic signals. A phase-1 trial evaluated the side effects secondary to dose escalation of magrolimab in patients with relapsed/refractory AML. While the patients remained asymptomatic, they all exhibited anemia due to red blood cell agglutination, as CD47 is expressed on red blood cells. Nonetheless, no hemolysis was described [142]. Magrolimab has the potential to work synergistically with tumor-targeting antibodies, as these antibodies can deliver an external pro-phagocytic signal via antibody-dependent cellular phagocytosis facilitated by macrophages [140]. In a phase-3 double-blind randomized trial involving around 432 unfit patients, the efficacy of the magrolimab–azacitidine–venetoclax combination was evaluated in comparison to magrolimab–azacitidine–placebo. Although phase-1 and -2 trials showed encouraging results, the study was halted due to an increased mortality risk, mainly secondary to infections and respiratory failure [143,144].

Evorpacept (ALX148) (ClinicalTrials.gov identifier: NCT04755244) targets CD47 and was administered in combination with azacitidine and venetoclax in a phase-1/2 clinical trial. The initial results show that an anti-leukemic effect was obtained, with a good safety profile. Further evaluation is necessary [145].

CC-90002, a CD47-SIRPα axis inhibitor, was administered as a single-agent therapy in patients with relapsed/refractory AML and high-risk MDS (ClinicalTrials.gov identifier: NCT02641002). However, the study was halted, as no relevant therapeutic response was observed [146].

Hu5F9-G4 was administered as a monotherapy or in combination with azacitidine, as a CD47 inhibitor, in a phase-1 clinical trial (ClinicalTrials.gov identifier: NCT03248479). This immune checkpoint inhibitor showed encouraging results, inducing a strong anti-leukemic effect with a good safety profile [147].

AUR103 calcium (ClinicalTrials.gov identifier: NCT05607199), TQB2928 (ClinicalTrials.gov identifier: NCT06008405), and AK117 (ClinicalTrials.gov identifier: NCT06387420) are other CD47 antagonists that are currently under evaluation in phase-1 and phase-1b/2 clinical trials, in association with azacitidine or azacitidine and venetoclax in patients with AML and MDS [148,149,150].

CD47’s overexpression in cancer cells makes it a compelling target for neoplastic disease treatment. Antibodies against CD47 have demonstrated promising results in AML/MDS treatment. Nevertheless, side effects linked to CD47-targeting treatments, such as cytopenia and hyperbilirubinemia, raise significant concerns that must be addressed [142].

Apart from those already mentioned, new immune checkpoint inhibitors are being investigated, such as bexmarilimab (BEX). This drug targets the scavenger receptor Clever-1, leading to enhanced antigen presentation, the release of pro-inflammatory proteins, and T-cell activation, showing promising results in solid tumor treatment [151]. Clever-1 is overexpressed on leukemic blasts and monocytes in patients with AML/MDS. In AML, BEX, as a single-agent therapy or in combination with azacitidine/venetoclax, enhances antigen presentation and activates T cells. In a phase-1 trial that enrolled 22 MDS/AML subjects who were refractory to hypomethylating agents, the BEX–azacitidine combination was well tolerated and demonstrated efficacy across various indications [152].

Recent studies suggest that in AML, macrophages might harbor leukemic mutations, which could affect their function and could represent potential therapeutic targets [153]. Thiostrepton, an antibiotic with antitumor effects on both hematological and non-hematological malignancies by targeting the PBX1-FOXM1 axis, was also found to modulate macrophage polarization [154,155,156]. Ex vivo experiments proved that thiostrepton induces apoptosis in the leukemic blast population and in the tumor-supportive macrophage–monocytic population in a dose-dependent manner [156]. Thiostrepton targets M1 and M2 macrophages; however, patients with high levels of M2 macrophages tend to benefit the most [156].

Rather than using macrophages as direct therapeutic targets, modulating their polarization yields promising results in cancer treatment and AML comprised. Macrophage receptors with a collagenous structure (MARCOs) are scavenger receptors that are highly expressed on TAMs and have been associated with a poor prognosis [157]. Eisinger and his colleagues altered macrophage polarization through anti-MACRO therapy, with the enhancement of NK-cell-dependent apoptosis [158]. In AML, high MACRO expression was linked to a high presence of M2 macrophages, suggesting that AML could benefit from anti-MACRO therapy [112].

TLRs activate innate immunity and polarize macrophages towards the M1 phenotype during pathogen invasion. In a phase-two clinical trial, Brenda J. Weigel et al. used TLR7 agonists to activate innate immune responses in patients with relapsed/refractory malignant hemopathies, including six with AML. However, the results were not spectacular, as most patients had progressive disease, while only one patient obtained a partial response, and one was considered as having stable disease [159]. Nonetheless, further research involving larger groups of patients is necessary to examine the impact of TLR agonists in AML.

Macrophage migration inhibitory factor (MIF) is an inflammatory protein, highly expressed in AML blasts and in the sera of AML patients [160,161,162,163]. Administered as single agents in AML, MIF inhibitors reprogram macrophages to switch their phenotype towards M1, and even more so in association with pro-inflammatory cytokines and CSF1R inhibitors [164]. Spertini et al. combined granulocyte–macrophage colony-stimulating factor (GM-CSF) with MIF inhibitors and observed a transition of M2 macrophages towards the M1 phenotype, leading to enhanced apoptosis and reversal of resistance to FLT3 and BCL-2 targeted therapies. Furthermore, the association of MIF inhibitors and GM-CSF in xenograft models results in a leukemia burden decrease [165]. No clinical trials are available for this therapy as of yet, but the results seem promising.

## 7. Conclusions

Due to their involvement in cancer progression and remarkable adaptability, targeting macrophages offers promising therapeutic options for neoplastic disease, hematological malignancies included. A more comprehensive understanding and characterization of macrophages associated with AML, both phenotypically and functionally, will aid in creating macrophage-targeted therapies that are more effective and less toxic. Future molecular strategies hold promise, such as monoclonal antibodies designed to modulate and enhance macrophage effectiveness against AML cells. The most promising approach to date focuses on the development of agents that target CD47 and CSF1R inhibitors. As of yet, despite some initial encouraging outcomes, infection and other potentially fatal complications outweigh the benefits. While the unfruitful results with magrolimab and pexidartinib may seem discouraging, new macrophage-targeting therapies are being explored and show promising results with potentially improved safety profiles. Furthermore, reprogramming macrophage polarization towards the pro-inflammatory subtype using MACRO and MIF inhibitors was associated with lower resistance to therapy. This suggests the potential benefit of creating synergies between existing treatments and macrophage reprogramming agents.

## Figures and Tables

**Table 1:** Macrophage differentiation and polarization.

Activated	Phenotype	The Main Secreted Cytokines	Function
LPS, IFN-γ	M1 macrophages	IL-1, IL-6, IL-8, IL-12, TNF-α, iNOS	Pro-inflammatory, Cytotoxic Antitumorigenic
IL-4, IL-13, IL-10, M-CSF	M2 macrophages		
	M2a	Il-10, CCL13, CCL17, CCL22	
	M2b	IL-10, CCL1, IL-12	Immunosuppressive Pro-tumorigenic Anti-inflammatory
	M2c	CCL16, CCL18	
	M2d	IL-10, IL-12, TGF-β, VEGF, CCL5, CXCL12, CXCL16	

Monocytes can transform into macrophages (M0), which may then further polarize into either an M1 or M2 phenotype. In response to LPS and IFN-γ, the M1 subtype releases pro-inflammatory cytokines while also producing iNOS, thus contributing to inflammation, cytotoxicity, and tumor destruction. Alternatively, monocytes can be polarized into M2 macrophages under the effect of IL-4 and IL-13. M2 macrophages secrete IL-10, TGF-β, and Arg1, which impart immunosuppressive and pro-tumorigenic effects. An imbalance between M1 and M2 macrophages creates a microenvironment conducive to tumor progression.
